# Hypothyroidism Versus Hyperthyroidism in Patients With Abnormal Uterine Bleeding: An Observational Study

**DOI:** 10.7759/cureus.116142

**Published:** 2026-09-12

**Authors:** Sheelu Sharma, Arpana Singhal, Reshu Gupta, Punam Harijan, Shubham Jaju, Aishwarya Mantri, Madhu Yasarapu

**Affiliations:** 1 Obstetrics and Gynaecology, Government Medical College, Chittorgarh, IND; 2 Obstetrics and Gynaecology, Rajasthan University of Health Sciences (RUHS) College of Medical Sciences, Attached to Government Rukmani Devi Beni Prasad (RDBP) Jaipuria Hospital, Jaipur, IND; 3 Physiology, Rajasthan University of Health Sciences (RUHS) College of Medical Sciences, Jaipur, IND; 4 Obstetrics and Gynaecology, Jhargram Government Medical College and Hospital, Jhargram, IND; 5 Pharmacology, Pacific Institute of Medical Sciences, Sai Tirupati University, Udaipur, IND; 6 Pathology, Pacific Institute of Medical Sciences, Sai Tirupati University, Udaipur, IND; 7 Pharmacology, Government Medical College, Suryapet, IND

**Keywords:** abnormal uterine bleeding, hyperthyroidism, hypothyroidism, menstrual disorders, thyroid dysfunction

## Abstract

Introduction

Abnormal uterine bleeding (AUB) is a common gynecological problem, and thyroid dysfunction is an important potentially reversible endocrine cause of menstrual abnormalities. Hypothyroidism and hyperthyroidism may produce different menstrual and clinical manifestations. The present study aimed to compare the clinical, menstrual, ultrasonographic, and laboratory characteristics of patients with hypothyroidism and hyperthyroidism presenting with AUB.

Materials and methods

A prospective observational study was conducted in the Department of Obstetrics and Gynaecology at Government RDBP Jaipuria Hospital, Jaipur, from January 2025 to December 2025 after Institutional Ethics Committee approval. A total of 252 women with AUB were enrolled using consecutive sampling. Participants underwent clinical, menstrual, ultrasonographic, and thyroid-function evaluation, with menstrual characteristics assessed at enrolment using a predesigned and structured questionnaire. Serum-free T3, free T4, and TSH were assessed using chemiluminescence immunoassay. Data were analyzed using IBM SPSS Statistics Version 26; categorical and continuous variables were compared using appropriate statistical tests, with p<0.05 considered significant.

Results

Among 252 women with AUB, hypothyroidism was identified in 33 (13.1%), while hyperthyroidism was present in eight (3.2%) women. Irregular menstruation was significantly more frequent among hyperthyroid patients (six (75.0%)) than hypothyroid patients (nine (27.3%), p=0.012), whereas menorrhagia was more frequent among hypothyroid patients (18 (54.5%)) than hyperthyroid patients (two (25.0%)). Lethargy and weight gain were observed in 22 (66.7%) and 15 (45.5%) hypothyroid patients, respectively, while weight loss occurred in eight (100.0%) hyperthyroid patients. Endometrial hyperplasia was observed in 15 (45.5%) hypothyroid and two (25.0%) hyperthyroid patients. Mean free T3, free T4, and thyroid-stimulating hormone (TSH) differed significantly between the groups (all p<0.001).

Conclusion

Hypothyroidism was more common than hyperthyroidism among women presenting with AUB and was predominantly associated with menorrhagia, whereas hyperthyroidism was more frequently associated with irregular menstruation and oligomenorrhoea. The contrasting clinical profiles support the importance of thyroid-function assessment in women presenting with AUB, particularly when characteristic menstrual or systemic features are present.

## Introduction

Abnormal uterine bleeding (AUB) is one of the most common gynecological complaints among women of reproductive age and encompasses abnormalities in the regularity, frequency, duration, or volume of menstrual bleeding [1]. Because AUB may arise from diverse structural and nonstructural causes, the International Federation of Gynecology and Obstetrics (FIGO) developed the PALM-COEIN classification to provide a standardized framework for describing and evaluating its etiologies [2,3]. Structural causes include polyp, adenomyosis, leiomyoma, and malignancy/hyperplasia, whereas nonstructural causes include coagulopathy, ovulatory dysfunction, endometrial disorders, iatrogenic causes, and causes not otherwise classified [4].

Endocrine disorders, particularly thyroid dysfunction, are important potentially reversible contributors to menstrual abnormalities [5]. Thyroid hormones have an important role in reproductive physiology, and abnormalities in thyroid function can interfere with the hypothalamic-pituitary-ovarian axis, ovulatory function, and endometrial physiology [6]. Consequently, both hypothyroidism and hyperthyroidism have been associated with abnormalities in menstrual patterns and reproductive function [7]. The clinical presentation may vary according to the type of thyroid dysfunction; hypothyroidism is frequently associated with heavy or prolonged menstrual bleeding, whereas hyperthyroidism may be associated with less frequent or irregular menstruation [8].

Studies evaluating thyroid status among women presenting with AUB have demonstrated variable distributions of thyroid abnormalities. In a descriptive cross-sectional study, Joshi et al. reported that most women with AUB were euthyroid, while hypothyroidism was more frequent than hyperthyroidism; hypothyroidism was identified in 11 (13.9%) patients and hyperthyroidism in one (1.2%) patient [9]. Menorrhagia was the most common bleeding pattern in that study. Similarly, another hospital-based study reported thyroid dysfunction among women presenting with AUB and highlighted the relevance of thyroid-function assessment in the evaluation of menstrual abnormalities [10]. These findings suggest that although thyroid dysfunction may represent a relatively small proportion of the overall etiological spectrum of AUB, its identification is clinically important because it represents a potentially treatable systemic cause [11].

Despite the established association between thyroid dysfunction and menstrual abnormalities, direct comparisons between women with hypothyroidism and hyperthyroidism presenting with AUB are relatively limited, particularly with respect to their demographic characteristics, menstrual patterns, clinical manifestations, ultrasonographic findings, and laboratory profiles. Therefore, the present study was undertaken to compare the demographic characteristics, menstrual patterns, clinical manifestations, ultrasonographic findings, and laboratory parameters among women with AUB diagnosed with hypothyroidism and hyperthyroidism. The study aimed to compare the clinical, menstrual, ultrasonographic, and laboratory characteristics of patients with hypothyroidism and hyperthyroidism presenting with abnormal uterine bleeding.

## Materials and methods

This prospective observational study was conducted in the Department of Obstetrics and Gynaecology at Government RDBP Jaipuria Hospital, attached to Rajasthan University of Health Sciences (RUHS) College of Medical Sciences, Jaipur, after obtaining approval from the Institutional Ethics Committee (IEC No. RUHS-CMS/Ethics Comm./2024/320). The study was conducted from January 2025 to December 2025 and included women from puberty to perimenopause presenting to the gynaecology outpatient department with AUB or menstrual disturbances. Eligible participants were recruited using consecutive sampling after obtaining written informed consent.

The sample size was calculated based on the previously reported prevalence of AUB of 17.9% in India, as reported by Shah et al. [11]. The required sample size was estimated using the single-population proportion formula:

$n = \frac{Z^2pq}{d^2}$

where n is the required sample size, Z=standard normal deviate at a 95% confidence level (1.96), p=anticipated prevalence of AUB (17.9%), q=1-p, and d=allowable absolute precision (5%). Based on these assumptions, the minimum required sample size was approximately 227 participants. After allowing for approximately 10% additional participants for possible incomplete data or loss to follow-up, the target sample size was approximately 250 participants. A total of 252 women with AUB fulfilling the predefined eligibility criteria were ultimately enrolled and evaluated during the study period. All participants underwent thyroid-function assessment, and those with thyroid abnormalities were categorized according to their thyroid status.

Women from puberty to perimenopause presenting with menstrual disturbances were eligible for inclusion. AUB was defined as bleeding from the uterine corpus that was abnormal in regularity, frequency, duration, and/or volume in a nongravid woman, in accordance with the International Federation of Gynecology and Obstetrics (FIGO) terminology for AUB [3]. The detailed inclusion and exclusion criteria used for participant selection are presented in Table 1.

**Table 1:** Inclusion and Exclusion Criteria The table presents the inclusion and exclusion criteria used for participant selection. Abbreviations: AUB, abnormal uterine bleeding; FIGO, International Federation of Gynecology and Obstetrics; IUCD, intrauterine contraceptive device.

Inclusion criteria	Exclusion criteria
Women from puberty to perimenopause presenting with menstrual disturbances	Women with palpable pelvic pathology, including fibroids, polyps, or cervical growths
Women presenting with abnormal uterine bleeding (AUB), defined as bleeding from the uterine corpus that was abnormal in regularity, frequency, duration, and/or volume in a nongravid woman, in accordance with FIGO terminology	Women with tuberculosis or other major systemic disorders
Women without hormonal preparation or thyroid replacement therapy	Women with diabetes mellitus or hypertension
Women with clinical features suggestive of hypothyroidism or hyperthyroidism	Women with coagulation abnormalities or bleeding diathesis
	Women using an intrauterine contraceptive device (IUCD) within the preceding two years
	Women receiving medications that could influence thyroid function or menstrual bleeding, including aspirin, heparin, sulpha drugs, antithyroid drugs, levothyroxine, glucocorticoids, or amiodarone

All eligible participants underwent a detailed clinical evaluation using a predesigned and structured questionnaire. Demographic characteristics, including age, occupation, marital status, and socioeconomic status, were recorded along with relevant past medical, family, and medication histories. Detailed menstrual history included the last menstrual period, cycle regularity, duration and pattern of bleeding, amount of blood loss, dysmenorrhoea, and duration of symptoms. General physical examination was performed to assess the overall condition and pallor, followed by abdominal, speculum, and pelvic examinations to evaluate uterine size, fornices, and other possible causes of AUB. Particular attention was given to clinical manifestations of hypothyroidism and hyperthyroidism, and a thyroid examination was performed. Routine laboratory investigations, urine pregnancy testing, and transabdominal ultrasonography were performed as clinically indicated.

For thyroid-function assessment, 5 mL of venous blood was collected in a plain, dry glass container without anticoagulant. Serum-free T3, free T4, and thyroid-stimulating hormone (TSH) levels were estimated using chemiluminescence immunoassay (CLIA) on the fully automated Access 2 Immunoassay System (Beckman Coulter, Brea, CA, USA) in the Biochemical Laboratory at RDBP Jaipuria Hospital. The laboratory reference ranges used for thyroid-function assessment were serum-free T4 60-120 ng/mL, serum-free T3 0.8-16 ng/mL, and serum TSH 0.5-5 mU/mL [12]. Participants were categorized as hypothyroid or hyperthyroid according to the thyroid-function test results and the predefined laboratory criteria. Patients identified with thyroid dysfunction were referred to the physician for further evaluation and management.

Data were entered and analyzed using IBM SPSS Statistics, Version 26 (IBM Corp., Armonk, NY). Categorical variables were summarized as frequency and percentage (n (%)), whereas continuous variables were expressed as mean±standard deviation (SD). Comparisons between the hypothyroid and hyperthyroid groups were performed using the chi-square test for categorical variables. Continuous variables were assessed for distributional assumptions and compared using the independent-samples t-test. A two-sided p-value <0.05 was considered statistically significant.

## Results

Among the total cohort of patients with abnormal uterine bleeding, hypothyroidism was identified in 33 (13.1%) patients, whereas hyperthyroidism was present in eight (3.2%) patients. Thus, hypothyroidism was more frequently observed than hyperthyroidism in the study population (Table 2).

**Table 2:** Frequency Distribution of Hypothyroidism and Hyperthyroidism Among Patients with Abnormal Uterine Bleeding (n=252) Data are presented as number (N) and percentage (%). Abbreviations: N, number of patients.

Thyroid dysfunction	N (%)
Hypothyroid	33 (13.1%)
Hyperthyroid	8 (3.2%)

The demographic characteristics showed that most patients in both groups were aged 21-30 years, comprising 16 (48.5%) patients in the hypothyroid group and four (50.0%) patients in the hyperthyroid group, followed by those aged 31-40 years, accounting for 15 (45.5%) and four (50.0%), respectively. Most patients belonged to the lower socioeconomic categories, with the lower socioeconomic group comprising 10 (30.3%) hypothyroid patients and two (25.0%) hyperthyroid patients. Regarding occupation, working women predominated in the hypothyroid group at 18 (54.5%), whereas working women and housewives were equally represented in the hyperthyroid group at four (50.0%) each. Most patients had normal BMI, observed in 17 (51.5%) hypothyroid and four (50.0%) hyperthyroid patients, while overweight was present in 13 (39.4%) and two (25.0%), respectively. Multiparous women constituted the majority of both groups, accounting for 22 (66.7%) hypothyroid and five (62.5%) hyperthyroid patients. None of the demographic or baseline characteristics demonstrated a statistically significant difference between the two groups (Table 3).

**Table 3:** Demographic and Baseline Characteristics of Patients With Hypothyroidism and Hyperthyroidism Data are presented as number (N) and percentage (%). The chi-square (χ²) test was used to compare categorical variables between the hypothyroid and hyperthyroid groups. A p-value <0.05 was considered statistically significant. Abbreviations: BMI, body mass index; N, number of patients; χ², chi-square; df, degree of freedom.

Variable	Category	Hypothyroid (n=33)	Hyperthyroid (n=8)	χ² (df)	p-value
Age group	<20	2 (6.1%)	0 (0.0%)	0.52 (3)	0.772
	21-30	16 (48.5%)	4 (50.0%)		
	31-40	15 (45.5%)	4 (50.0%)		
	>40	0 (0.0%)	0 (0.0%)		
Socioeconomic status	Upper	4 (12.1%)	0 (0.0%)	8.10 (4)	0.088
	Upper middle	6 (18.2%)	2 (25.0%)		
	Lower middle	4 (12.1%)	4 (50.0%)		
	Upper lower	9 (27.3%)	0 (0.0%)		
	Lower	10 (30.3%)	2 (25.0%)		
Occupation	Working	18 (54.5%)	4 (50.0%)	2.53 (2)	0.283
	Housewife	9 (27.3%)	4 (50.0%)		
	Student	6 (18.2%)	0 (0.0%)		
BMI, kg/m²	Underweight (<18.5)	2 (6.1%)	2 (25.0%)	2.98 (3)	0.395
	Normal weight (18.5-24.9)	17 (51.5%)	4 (50.0%)		
	Overweight (25.0-29.9)	13 (39.4%)	2 (25.0%)		
	Obesity (≥30)	1 (3.0%)	0 (0.0%)		
Parity	Nulliparous	4 (12.1%)	1 (12.5%)	0.78 (2)	0.677
	Primiparous	7 (21.2%)	2 (25.0%)		
	Multiparous	22 (66.7%)	5 (62.5%)		

Menstrual characteristics differed between the hypothyroid and hyperthyroid groups. Regular menstrual cycles were observed in 24 (72.7%) hypothyroid patients compared with two (25.0%) hyperthyroid patients, whereas irregular cycles were more frequent among hyperthyroid patients, occurring in six (75.0%) compared with nine (27.3%) hypothyroid patients; this difference was statistically significant (p=0.012). Regarding bleeding pattern, menorrhagia was the predominant presentation among hypothyroid patients, occurring in 18 (54.5%), whereas oligomenorrhoea was more frequent among hyperthyroid patients, occurring in four (50.0%). Polymenorrhoea was reported in four (12.1%) hypothyroid patients and was not observed among hyperthyroid patients, while dysmenorrhoea occurred in four (12.1%) and two (25.0%), respectively. The overall distribution of bleeding patterns did not differ significantly between the groups (p=0.141) (Table 4).

**Table 4:** Menstrual Characteristics of Patients with Hypothyroidism and Hyperthyroidism Data are presented as number (N) and percentage (%). The chi-square (χ²) test was used to compare categorical menstrual characteristics between the hypothyroid and hyperthyroid groups. A p-value <0.05 was considered statistically significant. Abbreviations: N, number of patients; χ², chi-square; df, degree of freedom.

Variable	Category	Hypothyroid (n=33)	Hyperthyroid (n=8)	χ² (df)	p-value
Menstrual regularity	Regular	24 (72.7%)	2 (25.0%)	6.32 (1)	0.012
	Irregular	9 (27.3%)	6 (75.0%)		
Bleeding pattern	Intermittent bleeding	2 (6.1%)	0 (0.0%)	6.90 (4)	0.141
	Polymenorrhoea	4 (12.1%)	0 (0.0%)		
	Menorrhagia	18 (54.5%)	2 (25.0%)		
	Dysmenorrhoea	4 (12.1%)	2 (25.0%)		
	Oligomenorrhoea	5 (15.2%)	4 (50.0%)		

Per-vaginal bleeding was the most frequently reported clinical manifestation in both groups, occurring in 26 (78.8%) hypothyroid patients and four (50.0%) hyperthyroid patients, followed by abdominal pain in 22 (66.7%) and four (50.0%), respectively. Loss of appetite was reported in 11 (33.3%) hypothyroid patients but was absent among hyperthyroid patients. Among physical signs, lethargy and weight gain were observed exclusively among hypothyroid patients, occurring in 22 (66.7%) and 15 (45.5%), respectively. In contrast, weight loss was present in eight (100.0%) hyperthyroid patients and absent among hypothyroid patients. Anxiety and restlessness were each reported in four (50.0%) hyperthyroid patients, while tremors were present in two (25.0%) patients. The differences in lethargy, weight gain, weight loss, anxiety, restlessness, and tremors were statistically significant (Table 5).

**Table 5:** Clinical Manifestations and Physical Signs Among Patients with Hypothyroidism and Hyperthyroidism Data are presented as number (N) and percentage (%). The chi-square (χ²) test was used to compare categorical clinical manifestations and physical signs between the hypothyroid and hyperthyroid groups. A p-value <0.05 was considered statistically significant. Abbreviations: N, number of patients; χ², chi-square; df, degree of freedom; NA, not applicable.

Clinical feature		Hypothyroid (n=33)	Hyperthyroid (n=8)	χ² (df)	p-value
Clinical manifestations	Per-vaginal bleeding	26 (78.8%)	4 (50.0%)	3.10 (1)	0.078
	Abdominal pain	22 (66.7%)	4 (50.0%)	0.72 (1)	0.396
	Loss of appetite	11 (33.3%)	0 (0.0%)	3.75 (1)	0.053
	Nausea/vomiting	0 (0.0%)	0 (0.0%)	NA	NA
Physical signs	Lethargy	22 (66.7%)	0 (0.0%)	10.47 (1)	0.001
	Weight gain	15 (45.5%)	0 (0.0%)	5.69 (1)	0.017
	Cold intolerance	7 (21.2%)	0 (0.0%)	2.10 (1)	0.147
	Weight loss	0 (0.0%)	8 (100.0%)	33.00 (1)	<0.001
	Anxiety	0 (0.0%)	4 (50.0%)	18.00 (1)	<0.001
	Restlessness	0 (0.0%)	4 (50.0%)	18.00 (1)	<0.001
	Tremors	0 (0.0%)	2 (25.0%)	8.25 (1)	0.004

Ultrasonographic abnormalities were relatively common among patients with hypothyroidism. Endometrial hyperplasia was the most frequent abnormality, observed in 15 (45.5%) hypothyroid patients compared with two (25.0%) hyperthyroid patients. Cystic ovaries were identified in 13 (39.4%) hypothyroid patients and two (25.0%) hyperthyroid patients, whereas a bulky uterus was observed in seven (21.2%) hypothyroid patients and was not observed among hyperthyroid patients. None of the evaluated ultrasonographic findings showed a statistically significant difference between the two thyroid dysfunction groups (Table 6).

**Table 6:** Abnormal Ultrasonographic Findings Among Patients with Hypothyroidism and Hyperthyroidism Data are presented as number (N) and percentage (%). The chi-square (χ²) test was used to compare categorical ultrasonographic findings between the hypothyroid and hyperthyroid groups. A p-value <0.05 was considered statistically significant. Abbreviations: N, number of patients; χ², chi-square; df, degree of freedom.

Abnormal ultrasonographic finding	Hypothyroid (n=33)	Hyperthyroid (n=8)	χ² (df)	p-value
Bulky uterus	7 (21.2%)	0 (0.0%)	2.10 (1)	0.147
Endometrial hyperplasia	15 (45.5%)	2 (25.0%)	1.04 (1)	0.308
Cystic ovaries	13 (39.4%)	2 (25.0%)	0.57 (1)	0.450

The mean hemoglobin concentration was lower among hypothyroid patients (10.9±2.4 g/dL) than among hyperthyroid patients (11.7±2.7 g/dL), although the difference was not statistically significant (p=0.446). The mean platelet count was higher in the hypothyroid group (168.3±33.7 ×10³/µL) compared with the hyperthyroid group (154.0±5.0 ×10³/µL), with a statistically significant difference (p=0.025). Thyroid hormone parameters showed marked differences between the groups, with lower mean T3 and T4 levels among hypothyroid patients (0.8±0.5 and 4.9±3.0, respectively) compared with hyperthyroid patients (2.7±0.4 and 15.5±0.9, respectively), while mean TSH was substantially higher among hypothyroid patients (48.6±30.8) than hyperthyroid patients (0.1±0.0); all three thyroid-function parameters demonstrated statistically significant differences (p<0.001) (Table 7).

**Table 7:** Laboratory Parameters Among Patients with Hypothyroidism and Hyperthyroidism Data are presented as mean±standard deviation (SD). The independent-samples t-test was used to compare continuous laboratory parameters between the hypothyroid and hyperthyroid groups. A p-value <0.05 was considered statistically significant. Abbreviations: T3, triiodothyronine; T4, thyroxine; TSH, thyroid-stimulating hormone; SD, standard deviation.

Laboratory parameter	Hypothyroid (n=33), Mean±SD	Hyperthyroid (n=8), Mean±SD	t-value	p-value
Hemoglobin (g/dL)	10.9±2.4	11.7±2.7	-0.77	0.446
Platelet count (×10³/µL)	168.3±33.7	154.0±5.0	2.33	0.025
T3	0.8±0.5	2.7±0.4	11.44	<0.001
T4	4.9±3.0	15.5±0.9	17.33	<0.001
TSH	48.6±30.8	0.1±0.0	24.32	<0.001

## Discussion

The present study compared the clinical and laboratory characteristics of women with AUB who had hypothyroidism and hyperthyroidism. Among the total study cohort, hypothyroidism was identified in 33 (13.1%) patients, whereas hyperthyroidism was present in eight (3.2%) patients, indicating that hypothyroidism was more frequently encountered. This pattern is broadly consistent with previous hospital-based studies of AUB. Joshi et al. reported that among women with AUB, 11 (13.9%) were hypothyroid and one (1.2%) was hyperthyroid, while Nekkanti et al. reported thyroid dysfunction in 15 (15.79%), with hypothyroidism accounting for nine (60.0%) and hyperthyroidism for two (13.33%) of thyroid-dysfunction cases [9,13]. The predominance of hypothyroidism in the present study therefore agrees with the existing literature. Most participants in both groups were aged 21-30 years, comprising 16 (48.5%) hypothyroid and four (50.0%) hyperthyroid patients, while multiparous women constituted 22 (66.7%) and five (62.5%), respectively. The concentration of cases in reproductive-age women is clinically relevant because thyroid dysfunction can influence ovulatory function and menstrual cyclicity [7].

The most clinically important finding was the difference in menstrual regularity and bleeding pattern between the two thyroid groups. Regular cycles were observed in 24 (72.7%) hypothyroid patients compared with two (25.0%) hyperthyroid patients, whereas irregular cycles occurred in nine (27.3%) and six (75.0%), respectively, with a statistically significant difference. Menorrhagia was the predominant bleeding pattern among hypothyroid patients, occurring in 18 (54.5%), while oligomenorrhoea was more frequent among hyperthyroid patients, occurring in four (50.0%) compared with five (15.2%) hypothyroid patients. These findings are consistent with the established association of hypothyroidism with heavier menstrual bleeding and hyperthyroidism with less frequent or reduced menstrual bleeding. In a tertiary-care AUB study, menorrhagia was the most common overall bleeding pattern, occurring in 34 (43.0%) patients [10]. Similarly, Güngör Semiz et al. found hypermenorrhoea to be significantly more common in overt hypothyroidism than in controls [14]. Conversely, hyperthyroidism has traditionally been associated with oligomenorrhoea and hypomenorrhoea, although the magnitude of menstrual disturbance may vary with disease severity [15].

The clinical phenotype in the present study also differed according to thyroid status. Lethargy and weight gain were observed in 22 (66.7%) and 15 (45.5%) hypothyroid patients, respectively, whereas weight loss was present in eight (100.0%) hyperthyroid patients; anxiety and restlessness were each reported in four (50.0%), and tremors in two (25.0%). These findings are consistent with the contrasting metabolic effects of reduced and excessive thyroid hormone activity and support the value of assessing thyroid-related symptoms in women presenting with menstrual abnormalities [8]. The ultrasonographic findings showed endometrial hyperplasia in 15 (45.5%) hypothyroid and two (25.0%) hyperthyroid patients, while cystic ovaries were observed in 13 (39.4%) and two (25.0%), respectively. Although these findings suggest potentially different reproductive tract profiles, the differences in individual ultrasonographic abnormalities were not statistically significant in the present comparison. Therefore, these findings should be interpreted cautiously, particularly given the relatively small hyperthyroid group. The absence of a significant difference also indicates that clinical thyroid status alone may not explain all structural ultrasonographic abnormalities observed in women with AUB.

Laboratory findings demonstrated the expected biochemical distinction between the two groups. Mean T3 and T4 concentrations were lower among hypothyroid patients (0.8±0.5 and 4.9±3.0, respectively) than among hyperthyroid patients (2.7±0.4 and 15.5±0.9, respectively), whereas mean TSH was substantially higher in hypothyroidism (48.6±30.8) than in hyperthyroidism (0.1±0.0), with all three parameters showing statistically significant differences (p<0.001). Hemoglobin was numerically lower in hypothyroid patients (10.9±2.4 g/dL) than in hyperthyroid patients (11.7±2.7 g/dL), although the difference was not statistically significant. The association between thyroid dysfunction and hematological abnormalities has been reported previously; a large pooled analysis found higher odds of anemia among individuals with overt hypothyroidism and overt hyperthyroidism compared with euthyroid participants [16]. A systematic review likewise described anemia as a frequent accompaniment of thyroid disease and highlighted the role of thyroid hormones in erythropoiesis [17]. Taken together, the present findings reinforce the clinical importance of evaluating thyroid function in women with AUB, particularly when menstrual patterns suggest hypothyroidism-associated heavy bleeding or hyperthyroidism-associated irregular or infrequent menstruation. However, the relatively small number of hyperthyroid patients warrants cautious interpretation, and larger comparative studies are required to establish the independent contribution of each thyroid disorder to specific AUB.

Strengths and limitations

The major strengths of the present study include its prospective observational design, systematic evaluation of women presenting with abnormal uterine bleeding, standardized assessment of menstrual characteristics and clinical manifestations, and objective confirmation of thyroid dysfunction using biochemical thyroid-function testing. The comparison of hypothyroid and hyperthyroid patients across demographic, menstrual, clinical, ultrasonographic, and laboratory parameters provides a comprehensive assessment of the differing clinical profiles of these disorders. However, the study has certain limitations. The relatively small number of patients in the hyperthyroid group may have limited the statistical power to detect differences between the two groups and warrants cautious interpretation of non-significant findings. The single-center design may also limit the generalizability of the findings to other populations and healthcare settings. In addition, potential residual confounding by unmeasured clinical, nutritional, hormonal, or socioeconomic factors cannot be excluded, and the observational design precludes establishing causal relationships between thyroid dysfunction and specific patterns of abnormal uterine bleeding.

## Conclusions

The present study demonstrates that hypothyroidism was more common than hyperthyroidism among women presenting with AUB, with distinct differences in their menstrual and clinical profiles. Hypothyroidism was predominantly associated with menorrhagia, whereas hyperthyroidism was characterized more frequently by irregular menstruation and oligomenorrhoea. Hypothyroid patients also showed typical manifestations such as lethargy and weight gain, while weight loss, anxiety, restlessness, and tremors were more prominent among hyperthyroid patients. Thyroid-function parameters showed marked biochemical differences between the two groups. These findings emphasize the importance of evaluating thyroid function as part of the assessment of women presenting with AUB, particularly when menstrual patterns or clinical features suggest an underlying thyroid disorder. Early recognition and appropriate management of thyroid dysfunction may help address potentially reversible causes of menstrual abnormalities and improve overall reproductive health outcomes.
